# The Importance of Context in Screening in Occupational Health Interventions in Organizations: A Mixed Methods Study

**DOI:** 10.3389/fpsyg.2017.01347

**Published:** 2017-08-09

**Authors:** Michela Vignoli, Karina Nielsen, Dina Guglielmi, Maria C. Tabanelli, Francesco S. Violante

**Affiliations:** ^1^Department of Education Studies, University of Bologna Bologna, Italy; ^2^Istituto Nazionale per la Valutazione del Sistema Educativo di Istruzione e di Formazione Rome, Italy; ^3^Management School, University of Sheffield Sheffield, United Kingdom; ^4^Department of Medical and Surgical Sciences, University of Bologna Bologna, Italy

**Keywords:** screening, psychological well-being, mixed methods, job demands-resources model, context

## Abstract

In occupational health interventions, there is a debate as to whether standardized or tailored measures should be used to identify which aspects of the psychosocial work environment should be targeted in order to improve employees’ well-being. Using the Job Demands-Resources model, the main aim of the present study is to demonstrate how a mixed methods approach to conducting screening enables the identification of potential context-dependent demands and resources in the workplace, which should to be targeted by the intervention. Specifically, we used a mixed methods exploratory sequential research design. First, we conducted four focus groups (*N* = 37) in a sample of employees working in grocery stores in Italy. The qualitative results allowed to identify one possible context-specific job demand: the use of a work scheduling IT software, whose implementation resulted in a high rotation between different market’s departments. From the qualitative results, this context-specific demand seemed to be related to workers’ well-being. Thus, in a subsequent questionnaire survey (*N* = 288), we included this demand together with generic measures of social support and psychological well-being. Results confirmed that this context-specific job demand was related to emotional exhaustion. Furthermore, it was found that social support moderated the relationship between this specific job demand and emotional exhaustion showing among employees whose activities depended on the IT software, employees that perceived higher levels of social support from colleagues experienced lower levels of emotional exhaustion with respect to their colleagues who perceived lower levels of social support. The present study confirms that mixed methods approach is useful in occupational health intervention research and offers a way forward on helping organizations prioritize their intervention activities.

## Introduction

For decades, studies have emphasized the role of work in determining psychological well-being (Cartwright and Cooper, 2008). According to the ‘Stress in America’ survey (American Psychological Association, 2014) work is the second most reported source of stress after money. Furthermore, both the European Union (Levi and Levi, 2000; European Agency for Safety and Health at Work [EU-OSHA], 2013) and the National Institute for Occupational Safety and Health (National Institute for Occuparional Safety and Health [NIOSH], 2015) have recognized the key role played by the psychosocial work environment in managing psychosocial risks and employees’ well-being. Psychosocial risk factors have been defined as ‘those aspects of work design and the organization and management of work, and their social and environmental contexts, which have the potential for causing psychological, social or physical harm’ (Cox et al., 2000, p. 14).

Occupational health interventions, which have been defined as “planned, behavioral, theory-based actions that aim to improve employee health and well-being” (Nielsen and Abildgaard, 2013, p. 278), are generally recommended because they target the sources of low psychological well-being (such as psychosocial risk factors), rather than the symptoms of low psychological well-being (International Labour Organization [ILO], 2001; European Agency for Safety and Health at Work [EU-OSHA], 2010). Despite these recommendations, there is still a long way to go. Considering stress as a condition of decreased well-being, a recent Cochrane systematic review conducted by Ruotsalainen et al. (2014) evaluated the effectiveness of occupational health interventions. They concluded that among the interventions considered there was little evidence of organizational interventions having an effect. Furthermore, the European Survey of Enterprises on New and Emerging Risks (European Agency for Safety and Health at Work [EU-OSHA], 2015) shows that although it is a legal requirement in the EU, only 3 out of 4 companies surveyed in the 28 European countries conducted a regular psychosocial risk assessment. In the European Countries, systematic approaches to manage employee well-being have been developed such as the Management Standards (United Kingdom), Work Positive (Ireland), START (Germany), SOBANE (Belgium), and INAIL-ISPESL (Italy) (Zoni and Lucchini, 2012). These methods employ a systematic approach where the intervention goes through the phases of initiation, screening, action planning, implementation, and evaluation. All of these methods recommend the use of standardized questionnaires that can be used across occupational and organizational settings for screening the psychosocial work environment.

Recently, standardized measures have been criticized for failing to consider the organizational context and therefore it may be difficult for organizations to develop suitable actions that target problem areas specific to the organization (Nielsen et al., 2014). In the present study, we focus on the screening phase because a thorough screening is crucial to ensure that suitable intervention activities that target the sources of low well-being are developed and implemented (Nielsen et al., 2014). Using a mixed methods approach, we first conducted focus groups to identify psychosocial risks specific to the local context and then test whether they are related to well-being outcomes in a survey. The study contributes to the existing literature in three ways. First, we address the call of Guglielmi et al. (2013) to understand how mixed methods design, i.e., combining quantitative and qualitative measures, could be useful in conducting context-specific screening for psychosocial risks. Second, a limitation of the above European methods to manage employee well-being is that they identify risks and build well-being profiles independently without considering the relationship between particular risks and well-being. We address the call of Nielsen et al. (2010a) and explore the interplay between the psychosocial work environment and well-being. Furthermore, the contribution of the study is to enhance knowledge concerning the intervention literature and particularly regarding the screening study.

### Theoretical Framework: Job Demands Resources Model

It is essential to rely on strong and validated theoretical models to understand the relationship between psychosocial risk factors and their consequences on employees (Nielsen et al., 2010b). The Job Demands-Resources (JD-R) model is one of the most widely used models concerning psychological well-being (Demerouti et al., 2001; Bakker and Demerouti, 2007; Bakker et al., 2014). The JD-R model posits that psychosocial risk factors can be divided into two categories: job demands and job resources. An important feature of the JD-R model is that demands and resources are not pre-defined, but should take into account the local context (Bakker et al., 2014). For example, pupil misbehavior could be a particular job demand for teachers (e.g., Bakker et al., 2007), or emotional labor for healthcare employees (e.g., de Jonge et al., 2008). Thus, the JD-R model is useful when assessing psychosocial risks in specific occupational groups as specific job demands and job resources could be specific to certain job positions (Bakker et al., 2014).

The JD-R model is based on two main propositions: the flexibility of the model and the existence of two processes, a health impairment and a motivational process (Bakker et al., 2014). The flexibility of the model relies in the fact that: (a) job characteristics can be modeled in job demands and job resources; (b) the model can be applied to all work environments and can be tailored to specific occupations. These characteristics of the JD-R model are really important in occupational health interventions as they allow researchers to conduct studies strongly based on the scientific literature, but also considering the real context where the studies are conducted.

There are two processes by which psychosocial factors may influence well-being. The health impairment process postulates that job demands could lead to employees’ depletion of energies and that demands constitute the most important antecedents of burnout. This process could also be related to different relevant outcomes, both health-related, such as depressive symptoms (e.g., Hakanen and Schaufeli, 2012) and job-related such as absenteeism (e.g., Vignoli et al., 2016). The motivational process states that job resources are the antecedents of work engagement. Moreover, one assumption of the JD-R model is that although job demands and job resources trigger different processes (i.e., the health impairment process and the motivational process), they also interact in predicting employees’ psychological well-being in two possible ways: (a) job resources could buffer the impact of job demands on burnout; (b) job demands could amplify the impact of job resources on work engagement (Bakker et al., 2014). Focusing on JD-R model could be useful in order to help organizations in designing interventions aimed to enhance employees’ well-being not only through the decreasing of job demands, but especially understanding the role of job resources, which interacting with job demands, could decrease the negative effects of these on psychological well-being (Bakker et al., 2014). In the present study, we operationalized psychological well-being using two outcomes: emotional exhaustion (the most central component in burnout) and work engagement in line with the JD-R model (Salanova et al., 2010).

### The Importance of Screening

Screening is an important part of occupational health interventions because it enables the identification of the risk factors on which to focus intervention activities (Nielsen et al., 2010a). Previous research has demonstrated how failing to develop activities that address the most pertinent problems perceived by employees may lead to the intervention failing to bring about the desired improvements in employee well-being (Aust et al., 2010).

Questionnaires can either be standardized to identify job demands and resources across occupational and organizational settings or tailored to the intervention in question (Nielsen et al., 2014). Both methods have advantages and disadvantages. The advantages of the standardized questionnaires are threefold. First, standardized questionnaires are generally recommended because they allow for comparisons across occupational groups and they allow for benchmarking within different groups of the same organization or different organizations (Nielsen et al., 2010a). Second, some demands and resources are believed to be non-specific, e.g., social support (Johnson and Hall, 1988). Thus, well-known psychosocial risk factors could be measured using standardized measures as they are already been used in organizational contexts. Finally, standardized measures have most often undergone rigorous validation procedures. A disadvantage is that for use in occupational health interventions, standardized questionnaires have drawbacks in that they are difficult to relate to the local context and work experiences of employees and thus are difficult to translate into concrete action plans (Nielsen et al., 2014). Likewise, tailored questionnaires have their advantages and disadvantages. Although some demands and resources (e.g., work pressure and autonomy) can be found in almost every occupational group, organizations have procedures or dynamics specific to them (Nielsen et al., 2014).

Tailored measures may enable researchers and occupational health practitioners to identify important context-dependent demands and resources. Tailored questionnaires capture the local context (Dewe, 1989) and can be used to develop detailed action plans that are meaningful to employees (Nielsen et al., 2010a). In a qualitative study, Nielsen et al. (2014) found that the use of a tailored questionnaire enabled participants to develop detailed and meaningful action plans, however, this method is not without its disadvantages. The disadvantages of the tailored questionnaires are that they do not allow for comparison across organizational settings and occupational groups and each tailored questionnaire needs their own thorough validation. Furthermore, one limitation of the Nielsen et al. (2014) study is that all demands and resources were measured using tailored items. This approach is very time-consuming and requires a high level of expertise in developing and validating questionnaires. As a result, the method may not be feasible in many organizations as researchers and occupational health practitioners may lack the expertise and time to develop questionnaires that only contain tailored measures. We argue that it is important only to use tailored measures to capture context-specific demands and resources that generic measures cannot capture. In the present study, we use the StART method (Guglielmi et al., 2013), which may combine the best of both worlds. It uses a mixed methods design to analyze both well-known demands and resources (e.g., workload and social support) and demands and resources specific to the organization. Mixed methods approach has been broadly defined as “*the type of research in which a researcher or a team of researchers combines elements of qualitative and quantitative approaches (e.g., use of qualitative and quantitative viewpoints, data collection, analysis, inference techniques) for the purpose of breadth of understanding or corroboration* (Burke Johnson et al., 2007, p. 123). Specifically, Following the research design proposed by Creswell and Plano Clark (2011), this study employs the *exploratory sequential design* first collecting and analyzing qualitative data (using focus groups) to gather useful information for developing a quantitative instrument (questionnaire) in order to test and generalize the qualitative findings in a tailored questionnaire. The purpose of this mixed methods research design is the need to test or measure qualitative exploratory findings (Creswell and Plano Clark, 2011).

Context has not been sufficiently recognized in the literature and it has been defined as “*situational opportunities and constraints that affect the occurrence and meaning of organizational behavior as well as functional relationships between variables”* (Johns, 2006, p. 386). Acknowledging the importance of considering the local context on the one hand but acknowledging the limitations of this method on the other, we used focus groups to identify risk factors that cannot be identified using standardized measures. To the best of our knowledge, the present study is the first to examine how a sequential mixed methods design can be used to develop context-specific items, that in combination with standardized scales to conduct screening of job demands and job resources can be used to inform the development of action plans to manage the psychosocial work environment and employee well-being. Mixed methods research designs have several advantages in identifying demands and resources in organizations. First, a mixed method approach ‘provides a better understanding of research problems, as only one type of data could provide an incomplete understanding’ (Creswell and Plano Clark, 2011, p. 5). This is because the mixed methods approach could overcome the limits of both qualitative and quantitative methods (Cortini, 2014).

This means that using only quantitative data or qualitative data could lead to the omission of important information about the context, e.g., important context-dependent demands and resources which cannot be detected using standardized quantitative measures only. Second, mixed methods, especially the sequential design, enable the researcher to generate and verify theories in the same study. This is possible because qualitative research can produce information about context-dependent demands and resources. It can then be tested whether these demands and resources are linked to well-being outcomes.

Finally, as Johnson and Turner (2003) argued that using mixed methods can balance out the drawbacks of each methods leading to a more in-depth analysis of the processes and mechanisms and the opportunities to compare quantitative data between different groups. For these reasons, mixed methods research may be suitable to develop screening tools that capture demands and resources specific to the local context.

## The Present Study

Given that tool oriented research in the organizational psychology field had not given enough attention to context (Johns, 2006) the main aims of the present study are (a) to demonstrate how mixed methods research can help identify context-specific job demands and resources; and (b) to analyze the links between job demands and job resources and psychological well-being. Such information is useful for organizations when they want to prioritize which actions to take to ensure employees well-being. In order to achieve these aims, a study was conducted in a large retail chain in Italy. Data were collected during the screening of psychosocial risk factors (demands and resources) in the company, an activity which is mandatory in Italy. As required by Italian law, different homogeneous groups (i.e., groups of employees who may be exposed to similar risks in the workplace) were identified. In the present study, we focus on the largest homogenous group composed by employees working in grocery stores. The qualitative and quantitative studies will be presented. Firstly, we used a qualitative approach as it allows to collect information about the context, which could not be easily gathered through standardized measures. From the qualitative results, potential context-specific demands and resources have been identified and hypotheses about how these context-specific demands and resources are linked to well-being outcomes have been developed. Subsequently, in the quantitative study, we tested whether these context-specific factors are related to well-being outcomes and how demands and resources may interact.

### Study 1: Qualitative Screening

#### Method

In this section, the qualitative study and its results will be presented. The main aim of this study was to identify potential context-specific demands and resources.

##### Participants and procedures

We gained access to the employee database to select a representative sample of employees based on the following information: age, organizational tenure, job position, and workplace (grocery store where the employees worked). Based on this dataset, a representative sample of 60 employees was identified and invited to participate in four focus groups (15 employees were selected for each focus groups). The recommended size for a focus group is between 10 and 12 people (Krueger and Casey, 2015). We expected drop-out due to maternity leave, sickness leave, incompatibility between focus groups scheduling and shift work of that week, or unwillingness to participate in the study. A general communication about the project was sent by e-mail to all the supervisors and, as many of the employees did not have access to e-mail, the communication was advertized on the bulletin boards in every grocery store. An invitation to participate was sent in an informative letter to their supervisor. Participation in the study was promoted by the Safety manager, and through telephone calls to supervisors a few days prior to the scheduled focus groups. The final sample included 37 participants (mean participants for focus group was 9.2), and the participation rate was 61.6%. Of these 62.2% were female, mean age was 44.6 years (*SD* = 8.87) and the average organizational tenure was 12.62 years (*SD* = 9.46). Focus group were recorded and then transcribed verbatim.

##### Measures

Four main parts composed the focus group’s structure. In the first part, the interviewer presented him/herself and the aim of the focus group and answering questions about the project. In the second part, the researcher read the definition of stress presented in the Framework Agreement on stress at work (ETUC, 2004). Subsequently, the interviewer invited the participants to identify demands and resources in their job thinking in the past 6–12 months. To ensure all major aspects of the psychosocial work environment had been covered, respondents were then probed about demands and resources using the European Agency for Safety and Health at Work as Stressful Characteristics of Work divided in two main categories: work content (work environment and work equipment, task design, workload/workplace and work schedule) and work context (organizational culture and function, role in the organization, career development, decision latitude/control, interpersonal relationship at work, home-work interface).

##### Data analysis

Two researchers analyzed data from focus groups with NVivo software (version 10) using the thematic analysis technique. A codebook was created and nodes were developed based on the Cox et al. (2000) categories of context of work (organizational culture and function, role in organization, career development, decision latitude/control, interpersonal relationships at work, home-work interface) and content of work (work environment and work equipment, task design, workload/workplace, work schedule). Researchers analyzed the text passages and assigned codes to statements which referred to the main job demands and job resources. In case of disagreement, the two researchers discussed until reaching consensus.

#### Qualitative Results

The thematic analysis identified the presence of a potential context-dependent job demand in the population. In fact, besides giving many information about the categories presented before, some employees mentioned an IT software called Brase. This software was mentioned in two out of the four focus groups, possibly because that not all grocery stores had implemented the software. Furthermore, in some stores not all the employees’ activities depended on the Brase IT software. Several issues were reported relating to having your work scheduled according to the Brase IT software, specifically workers reported the following statements: “I have never had stress problems but, with Brase you stay 2 h in the butcher’s department, 1 h in the gastronomy department and so on. It is stressful because I change too much, it makes me crazy”; “I have to go to other departments and I’m not able to do the job”; “The aim of Brase is optimizing times, but without knowing what to do, we have the double amount of work”; “Brase produced confusion, it’s the second time I caught the flu. I stayed in the bakery department and then in the fish department and then you got sick. Basically, we did something similar also before but not to the same extent”; “Brase is the first cause of stress, I begin ten activities and in the end neither is performed well. It makes us feel frustrated and there is no satisfaction in working in this way.” Furthermore, the Brase IT software was discussed in relation to the working schedule. One worker reported that: ‘We just have work shifts that you know when you enter but you don’t know when you are going to quit, and, in addition to this, they change our work shifts under the wire. In addition, with Brase they (the coordinators) took half of a day to organize the working hours and they give it to you on Saturday for Monday.’ Moreover, it was also mentioned that: ‘Top management should be closer to employees. Take for example Brase, if they had asked for our collaboration, then it would be different.’ One of the company manager confirmed us that Brase had been developed to optimize time and enhancing the organization’s productivity through enabling the individual grocery store’s coordinator to organize the employees’ tasks more efficiently. The consequence of the implementation of the Brase software was a high rotation between different market’s departments and consequently the IT software was perceived by participants to have a negative influence on their well-being. In conclusion, participants perceived the Brase software to be a demand in their jobs that was associated with low well-being. The information about the local context dependent IT software was fed into questionnaire development. In line with the exploratory sequential design, we used the input from the qualitative study to inform the questionnaire. To ensure a high response rate in a sample of employees with little formal education, we decided to only include one item on Brase, which was ‘Do your work activities and the planning of them depend on ‘Brase’ software?’

### Study 2: Quantitative Screening

#### Method

This translation of qualitative results into a one-item measure in the questionnaire, allowed us to test: (a) the hypothesis that the Brase IT software was as a job demand that was related to poor well-being and (b) whether any resources in the grocery stores could buffer the negative impact of the Brase IT software on employees’ well-being. Testing these two hypotheses provides us with important information on which demands and resources organizations need to prioritize in the development of subsequent action plans.

We suggest employees working in grocery stores employing the Brase IT software may perceive Brase as a job demand because our qualitative findings suggested that after the implementation of Brase, employees reported their working conditions had worsened. As the health-impairment process of the JD-R model postulates that job demands are related to emotional exhaustion, we propose that the problems associated with the Brase IT software may result in it being related to emotional exhaustion, i.e., that employees reported it took them twice as long to do the job and employees did not know how to perform new job tasks in sections of the grocery store they had not previously worked in and these issues are likely to be related to emotional exhaustion. In order to test and generalize the qualitative results and according to the JD-R model we formulated the following hypothesis:

Hypothesis 1: We expect that employees whose working time is scheduled using the Brase IT software will report higher levels of emotional exhaustion.

Employees who have to deal with Brase reported to experience frustration and confusion as they are not able to do the job. For this reason, we hypothesize that Brase would be negatively associated with work engagement. Although the original JD-R model suggests that job demands are related to burnout and not work engagement, scholars have called for the need to understand the relationship between job demands and work engagement. Specifically, Schaufeli and Taris (2014) in their recent critical review of the JD-R model suggest that future research should investigate the direct and indirect effect of job demands on work engagement. Furthermore, the Brase IT software could affect the meaning of work perceived by the employees, as they have to change too many activities without clear goals. In fact, meaning of work refers to ‘finding a purpose in work that is greater than the extrinsic outcome of the work’ (Arnold et al., 2007, p. 195). In line with this, meaning of work has been found to be related to work engagement in recent studies (e.g., Beukes and Botha, 2013; Ghadi et al., 2013). Thus, we developed the following hypothesis:

Hypothesis 2: We expect that employees whose working time is scheduled using the Brase IT software will report lower levels of work engagement.

As the economic crisis and the increasingly complex labor markets put pressure on organizations, it may not be feasible to reduce employees’ job demands (for example reducing the amount of work). In the case of the Brase IT software, the company had introduced the IT software with a view to reduce costs. It was therefore not feasible to recommend the Brase software be abandoned. As suggested by Bakker et al. (2014) in their review of the JD-R model, interventions should aim to prevent burnout and foster work engagement through improving resources at work, especially when it is not feasible to decrease the level of job demands. As previously reported, job demands and job resources trigger different processes (i.e., the health impairment and the motivational processes), but they may also interact. First, job resources may buffer the impact of job demands on emotional exhaustion. Bakker and Demerouti (2007) proposed that job resources relating to the interpersonal and social relations are important in ensuring worker well-being. In the present study, we propose that because employees working according to the Brase IT software reported had to cope with ‘not being able to do the job,’ ‘not knowing what to do,’ thus social support may function as a valuable job resource able to buffer the negative impact of working in grocery stores that had implemented the IT software. Employees who seek information about how to do the jobs within the retail store from colleagues or could count on colleagues’ support, may be better able to cope with the demands created by the rotation between jobs. Many studies confirmed the moderating role of social support in the relationship between job demands and well-being outcomes as research on social support demonstrated its ability to moderate against job stress (Bakker et al., 2004). For example, Demerouti et al. (2011) demonstrated that social support buffers the relationship between work-family conflict and absenteeism. Moeller and Chung-Yan (2013) found that social support moderates the relationship between amounts of work and decrease the level of related job stress. Similarly, Xanthopoulou et al. (2007) found that social support moderated the relationship between workload and emotional exhaustion. Therefore, we formulated the following hypothesis:

Hypothesis 3: We expect that being a Brase worker results in lower levels of emotional exhaustion for employees that perceive high levels of social support from colleagues.

We propose that employees whose work schedule is organized with the Brase IT software and who experience high levels of support will report lower levels of exhaustion than those who report low levels of support. Job demands and job resources may also interact to predict work engagement. It is possible that employees who employ social resources such as the support of others to deal with the demands of the job may become more engaged with their job because they feel they are more capable of doing the job and thus they feel able to dedicate themselves to the task at hand. They may also come to feel energized through the interaction with colleagues to solve any problems created by the Brase IT software such as working in parts of the grocery store where they do not know how to do the job. Bakker et al. (2007) found in a sample of teachers, supervisor support moderated the relationship between pupil misbehavior and work engagement, such that teachers who experienced pupil misbehavior were more engaged in their job if their supervisor supported them. We therefore propose a fourth Hypothesis:

Hypothesis 4: We expect that being a Brase Worker results in higher levels of work engagement for employees that perceive high levels of social support from colleagues.

We propose that employees whose work schedule is organized according to the Brase IT software will report higher levels of work engagement if they feel supported by their colleagues.

##### Participants and procedures

In total, 107 grocery stores participated in the study. Each grocery store had an average of 27 employees. For participation in the survey part of the screening, a representative sample of 775 grocery shop workers were identified based on organizational personnel records. We selected the sample using the following criteria: age, organizational tenure, job position, and workplace. In order to test and generalize the qualitative findings and avoid bias, employees who had attended a focus group were not invited. Participants convened in group meetings lasting approximately 2 h. In the first part of these meetings, the researcher explained the national regulation concerning health and well-being at work and the project that involved that company. Then employees completed the questionnaire. A researcher was present during the session in case participants needed more information. Participants in the survey were invited through an informative letter sent to their supervisor. Participants totalled 551 (response rate = 71.1%) and worked in grocery stores of different sizes: 35.2% worked in big grocery stores (3,500–15,000 sqm of sales area); 52.3% worked in medium grocery stores (2,000–3,500 sqm of sales area); 12.5% worked in small grocery stores (0–1,000 sqm of sales area). This distinction created by the company is relevant as it results also in different procedures among the grocery stores. The Brase IT software had only been introduced in medium grocery stores and therefore we only included employees who from these stores in our analyses. The final sample of the quantitative study was 288. Most of the participants (70.5%) were female and the mean age was 46.3 years (*SD* = 7.7). Organizational tenure mean was 19.1 years (*SD* = 8.8) and the mean of working hours in a week was 31.4 h (*SD* = 5.9). Most of the participants (90.6%) had permanent contracts. Ethical approval was not required for this study in accordance with the national and institutional guidelines.

##### Measures

In line with the focus group, we developed a general questionnaire composed of many scales related to the content and context factors (Cox et al., 2000) categorized into job demands and job resources. The final questionnaire was very long (133 items). More specifically, in order to test our study hypotheses, we included validated measurement of social support from colleagues, emotional exhaustion and work engagement. Furthermore, according to the mixed methods exploratory sequential design and the qualitative results, we developed one item concerning the Brase software.

*Being a Brase-worker*. One item was developed based on the results of the focus groups: ‘Do your work activities and the planning of them depend on ‘Brase’ software?’ Responses to this item were dichotomous (‘yes’ or ‘no’).

*Social support from colleagues*. To investigate this job resource, four items from scale Karasek’s (1985; Italian version: Cenni and Barbieri, 1997, Unpublished) have been used. This standardized scale was chosen because social support is a generic resource (Johnson and Hall, 1988). The scale is a four-point Likert scale, ranging from ‘1’ (definitely not) to ‘4’ (decidedly). One example item is ‘People I work with are helpful in getting the job done.’ Cronbach’s alpha was 0.71.

*Emotional exhaustion*. Emotional exhaustion was measured with the emotional exhaustion dimension of the MBI-General Survey (Schaufeli et al., 1996; Borgogni et al., 2005). One example item is ‘I feel emotionally drained from my work.’ The 5-item scale was scored on a 7-point frequency scale (0 = never to 6 = every day). Cronbach’s alpha was 0.81.

*Work engagement*. The short nine-tem version of the Utrecht Work Engagement Scale (Schaufeli et al., 2006; Italian version: Balducci et al., 2010) was used, consisting of three for each dimension: vigor, dedication, and absorption. One example item is ‘At my job, I feel strong and vigorous.’ All the items related to dimensions of work engagement were scored on a seven-point scale ranging from ‘0’ (never) to ‘6’ (always). Cronbach’s alpha was 0.87.

Moreover, five control variables, which could potentially be correlated with psychological well-being outcomes were introduced: gender (e.g., Purvanova and Muros, 2010), age (e.g., Ahola et al., 2008), organizational tenure (e.g., Van Dam et al., 2008), working hours (e.g., Leonardi et al., 2013) and type of contract (e.g., Pirani and Salvini, 2015).

##### Data analysis

Statistical analyses were performed using SPSS version 20. At a first step, control variables, which showed a significant correlation with our well-being outcomes, were entered. To test the hypotheses, hierarchical regressions were used. In step 2, Brase and social support were introduced testing hypotheses 1 and 2. In the last step, the interaction term between Brase and social support was introduced in order to test hypotheses 3 and 4. Both the independent and the moderator variable were mean-centered.

#### Quantitative Results

Results presented in **Table 1** shows descriptive statistics and correlations between variables.

**Table 1:** Means, standard deviations, reliabilities, and Pearson correlations for all variables (*N* = 288).

Variables	*M*	*SD*	1	2	3	4	5	6	7	8
(1) Gender^a^	0.29	0.46	–							
(2) Age	46.33	7.67	-0.13*	–						
(3) Tenure	19.11	8.85	-0.03	0.64***	–					
(4) Working hours	31.41	5.95	0.42***	0.16**	0.25***	–				
(5) Contract^b^	0.91	0.29	-0.05	-0.13*	-0.08	-0.06	–			
(6) Brase^c^	0.40	0.49	-0.02	0.04	0.04	0.02	0.05	–		
(7) Social support	2.88	0.62	0.04	-0.05	-0.01	0.05	0.01	0.05	–	
(8) Exhaustion	18.65	7.59	-0.13*	0.21***	0.24***	-0.02	0.05	0.14*	-0.28***	–
(9) Engagement	4.80	1.13	-0.01	-0.07	-0.18**	-0.06	-0.08	-0.03	0.37***	-0.40***

^∗^*p < 0.05, ^∗∗^*p* < 0.01, ^∗∗∗^*p* < 0.001, ^a^Male = 1; 28.7%, ^b^Tenured = 1; 92%, ^c^Brase yes = 1; 22.9%*.

Concerning the 288 participants, 116 (40.3%) had their work organized by the Brase IT software. Results of the Pearson correlations showed that having your work scheduled by the Brase IT software is positively related to emotional exhaustion (*r* = 0.14, *p* < 0.05), but not to work engagement (*r* = -0.02, *p* > 0.05), social support (*r* = 0.05, *p* > 0.05). The relationships between social support and outcome variables are in the expected direction: social support is positively related to work engagement (*r* = 0.37, *p* < 0.001) and negatively related to emotional exhaustion (*r* = -0.28, *p* < 0.001). As working hours and type of contract are not related to emotional exhaustion and work engagement, they were excluded from the regression analyses. Results presented in **Table 2** support Hypothesis 1: Brase was positively related to emotional exhaustion (β = 0.15, *p* < 0.01). Brase and work engagement were not related (β = -0.04, *p* > 0.05), thus Hypothesis 2 was rejected.

**Table 2:** Interaction effect between Brase and social support from colleagues on emotional exhaustion and work engagement (*N* = 288).

	Emotional exhaustion	Work engagement
**Step 1: Control variables**		
Gender^a^	-0.13^∗^	-0.01
Age	0.08	0.06
Tenure	0.18^∗^	-0.22^∗∗^
*R*^2^	0.08^∗∗∗^	0.03^∗^
**Step 2: Main effects**		
Brase^b^	0.15^∗∗^	-0.04
Social support	-0.29^∗∗∗^	0.38^∗∗∗^
Δ*R*^2^	0.10^∗∗∗^	0.15^∗∗∗^
**Step 3: Two-way interaction**		
Brase × Social support	-0.16^∗^	0.13
Δ*R*^2^	0.01^∗^	0.01

^a^*Male = 1; ^b^Brase yes = 1, ^∗^*p* < 0.05, ^∗∗^*p* < 0.01, ^∗∗∗^*p* < 0.001. β-values are presented*.

**FIGURE 1:**
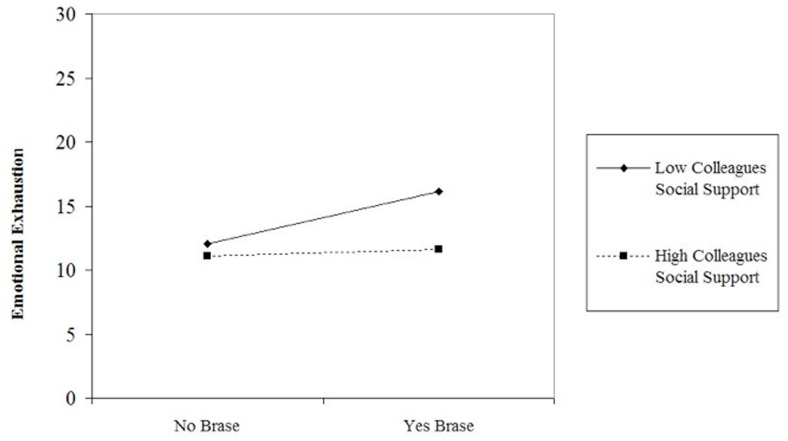
Moderation of social support from colleagues on emotional exhaustion.

Concerning the first moderation Hypothesis, our third Hypothesis, social support from colleagues moderated the relationship between Brase and emotional exhaustion (β = -0.16; *p* < 0.05, Δ*R*^2^ = 0.014). The direction of this relationship reported in **Figure 1** indicates that Brase employees who perceived higher levels of social support from co-workers experienced lower levels of emotional exhaustion with respect to their colleagues who perceived low social support. Thus, Hypothesis 3 was supported. Moderation analysis shows that social support from colleagues does not moderate the impact of Brase on work engagement (β = 0.13; *p* > 0.05, Δ*R*^2^ = 0.01), thus Hypothesis 4 was rejected. Furthermore, social support affects work engagement (β = 0.38; *p* < 0.001).

## Discussion

In the present study, we demonstrated how a mixed methods approach (in particular the exploratory sequential design) may be suitable for identifying the demands and resources that organizations need to address to ensure employee well-being. Using focus groups, we identified a context-dependent job demand: organization’s use of a IT software to schedule employees’ work in different departments of the grocery stores. In a quantitative survey, we confirmed that employees working under this IT software reported higher levels of emotional exhaustion, thus confirming what has been suggested from the qualitative results, which was that the Brase IT software was a contextual job demand. These results jointly gave many information both from a quantitative prospective (data supported the effects of the specific job demand) and from a qualitative perspective (results from the focus group suggested how this specific job demands could interfere with the workers’ well-being) indicated that the organization should prioritize planning activities to address the use of this IT software to schedule employees’ work. The review of Ruotsalainen et al. (2014) revealed that interventions to change working schedules had little effect. Our study provides valuable information as to what the issue was concerning working schedules, i.e., that these were planned using an IT software program that brought about a range of problems. Such detailed information may help organizations develop interventions address the underlying problems.

In light of the contextual constraints that the grocery stores face it would not be feasible for them to abandon the IT software altogether and we therefore tested whether social support be a resource that could protect against the negative impact of having your work scheduled according to the Brase IT software. We found that employees who work under the Brase IT software and who experience low support report higher levels of exhaustion. We thus found the mixed methods exploratory sequential design may be a useful approach to identify job demands and job resources in occupational health interventions. Our mixed methods research design allows for first exploring the organizational context and dynamics qualitatively identifying context-specific job demands and job resources, and then quantitatively verifying findings thus enabling generalization to the broader working population.

### Major Findings and Theoretical Implications

In the first study, focus groups were conducted, during which employees identified a context-dependent job demand, i.e., a IT software called Brase that was used to schedule work activities and rotas. The qualitative findings showed that Brase was associated with different kinds of problems (e.g., ‘confusion,’ ‘lack of clarity,’ ‘work overload,’ ‘frustration,’ ‘not being able to do the job to a satisfactory standard,’ ‘not knowing how to do the job’ etc.). Qualitative findings allowed us to design the subsequent quantitative study in which it has been hypothesized that Brase could be considered a job demand and thus, that employees would experience higher level of emotional demands and lower levels of work engagement if they reported having their work scheduled by the Brase IT software. Concerning the first two hypotheses which suggested an association between having your work scheduled according to the Brase IT software and psychological well-being (i.e., emotional exhaustion and work engagement), findings showed that Brase was positively related to emotional exhaustion (Hypothesis 1), but not work engagement (Hypothesis 2). The positive association between Brase and emotional exhaustion support the JD-R model suggesting that the main antecedents of burnout are job demands (Bakker and Demerouti, 2007; Bakker et al., 2014) as demonstrated also by the meta-analysis of Lee and Ashforth (1996), which showed that job demands are the most important predictors of burnout. Thus, findings suggest that Brase could be considered as a job demand as it is related only to emotional exhaustion and not to work engagement. Concerning the moderation hypotheses (Hypotheses 3 and 4), findings indicated that social support moderated the relationship between Brase and emotional exhaustion. Particularly, this means that Brase-employees who experience higher levels of social support report lower levels of emotional exhaustion compared to Brase-employees with lower levels of social support. The interaction was such that low levels of social support were related to high levels of emotional exhaustion among employees working according to the Brase IT software. This result is in line with the JD-R model suggesting that social support is an important moderator of the relationship between job demands and emotional exhaustion (e.g., Xanthopoulou et al., 2007). No support was found for the moderation of social support from colleagues on work engagement, despite this interaction is one of the two possible interaction processes proposed in the JD-R model. This result is in line with the health impairment process, which posits that job demands are related to burnout and not to work engagement. This was suggested by the lack of support for Hypothesis 2.

### Practical Implications

Findings of the present study offer some suggestions concerning how to use mixed methods exploratory sequential design in order to understand how we may identify context-specific job demands and resources. The combined use of qualitative and quantitative approach provides a reliable way for organizations not only to detect context-specific job demand and resources in a particular population but it could also provide useful suggestions on how to develop reliable and effective intervention activities modeled on specific groups of employees in the organization in order to enhance well-being. The results of the present study suggested that in a context where it may not be feasible to minimize job demands as the context-dependent job demand detected (i.e., having your work organized by the Brase IT software) was an essential part of the organization’s strategy to decrease costs associated to human resources increasing the resource of social support may be an effective strategy to minimize employees’ emotional exhaustion. An effective strategy for reducing the negative impact of having work scheduled by the Brase IT software may be to develop intervention activities focused on increasing social support. Such activities could include introducing a buddy system, where employees who were allocated to new departments of the supermarket would be allocated ‘a buddy’ who could show them the ropes. Another option could be to name a group of expert employees in each area of the supermarket and make sure that (1) on each shift one such employee was at work in that department and (2) new employees knew whom they could contact for support.

### Strengths and Limitations and Suggestions for Future Research

The main strengths of the present study are the sequential mixed methods design that allowed us to identify context-specific job demands, the high participation and response rates (61.7% in qualitative study and 71.1% in quantitative study), and the use of two different samples for the qualitative and the quantitative studies of the project. Some limitations of the study have to be acknowledged in interpreting the results. First, only one context-specific item was developed on the basis of the qualitative findings and this could affect the validity of this research, as this item may not capture the range of issues related to the use of the IT software. However, as the item developed is context-dependent, and it is not measuring a psychological construct, but whether employees working in retail store that has installed the Base software, we argue the measure is sensitive to capture the essence of the demand. Organizations would be able to go back to the qualitative data to gain more information about the specific issues concerning the Brase IT software that might help them develop supportive actions. The quantitative study used self-report only data and no objective measures were included. It has been argued that issues related to common-method variance may have been overestimated in organizational research (Spector, 2006). Furthermore, our focus was on employees’ own perceptions and experiences rather than objective measures of the constructs studied and participants rated different aspects, i.e., the use of an IT software, their perceptions of support from others and their own well-being. Social support and well-being constructs would be very difficult to measure with objective data in a reliable way. For practical reasons, we were unable to obtain objective measures from the organization on the use of the Brase IT software as the organization was mainly interested in the costs concerning the amount of reduced working hours, not the actual use of the program. In addition, the quantitative section of this study is cross-sectional, thus it is not possible to draw inferences about causality. As the present study focuses on the screening phase of an intervention, it is important that feedback is provided shortly after data collection to keep up momentum. Future research should consider all the phases of occupational health interventions to understand whether the use of tailored measure enable the development and implementation of intervention activities that are successful in improving the psychosocial work environment and employee well-being.

## Conclusion

The contributions of the present study are twofold. We contribute to knowledge on (a) how to design screening in occupational health interventions considering the local context; (b) propose ways forward for analyzing psychosocial risk screening data to help organizations in taking appropriate action to manage their psychosocial work environment and the psychological well-being of employees. First, using a sequential mixed method design combined with both tailored and standardized measures may enable organizations to develop and implement intervention activities relevant to the local context. The present study highlights the importance of combining tailored and standardized measures to conduct screening and presents a way to integrate the organizational context into such screening. In this way, organizations could effectively screen their own psychosocial work environment in a sensitive and cost-effective way. Second, based on a strong theoretical framework (the JD-R model) we explored how the interaction between job demands and resources may be useful for organizations that aim to enhance employees’ well-being through increasing resources when it is not feasible to decrease job demands.

## Author Contributions

MV, DG, MT, and FV conceptualized the mixed method study and chose the theoretical framework and they define the tools used. MV and MT collected the data. MV performed the data analyses and wrote the first draft of the paper. KN revised the paper for methodological and theoretical content. MV wrote the final version of the paper accordingly to KN, DG, MT, and FV critical revisions of the work for important intellectual content. All the authors gave final approval of the paper.

## Conflict of Interest Statement

The authors declare that the research was conducted in the absence of any commercial or financial relationships that could be construed as a potential conflict of interest.
